# The gut-brain axis in Alzheimer’s and Parkinson’s diseases: a comparative perspective

**DOI:** 10.3389/frmbi.2026.1942228

**Published:** 2026-09-30

**Authors:** Riddhi Upadhyay, Chandhini C., Susmita Kar, Sajith Sathyamoorthy, Xavier Pitchaimuthu, Murugan Sevanan

**Affiliations:** 1 Division of Biotechnology, Karunya Institute of Technology and Sciences (Deemed to be University), Coimbatore, India; 2 Department of Maths, Karunya Institute of Technology and Sciences (Deemed to be University), Coimbatore, India

**Keywords:** Alzheimer’s disease, artificial intelligence and machine learning, gut microbiota dysbiosis, gut-brain axis, Parkinson’s disease

## Abstract

Global health challenges associated with neurodegenerative disorders are increasing, prompting growing interest in the gut-brain axis (GBA) as a key contributor to disease pathogenesis. The GBA represents a bidirectional communication network linking the gut microbiota, immune signalling pathways, metabolic processes, and the central nervous system (CNS). This review highlights the role of the GBA in the onset and progression of Alzheimer’s disease (AD) and Parkinson’s disease (PD), focusing on comparative disease mechanisms involving microbial dysbiosis, neuroinflammation, and metabolic dysfunction. Particular emphasis is placed on alterations in short-chain fatty acids (SCFAs), lipopolysaccharide (LPS), microbial amyloids, and inflammatory mediators including tumour necrosis factor alpha (TNF-α), interleukin 1 beta (IL-1β), interleukin 6 (IL-6), toll-like receptor 4 (TLR4)/nuclear factor kappa B (NF-κB), and NLR family pyrin domain containing 3 (NLRP3) inflammasome, which contribute to protein misfolding, blood-brain barrier disruption, and the accumulation of amyloid-β, hyperphosphorylated Tau, and α-synuclein. Furthermore, the review discusses recent advances in artificial intelligence (AI) and machine learning (ML) for integrating microbiome, clinical, neuroimaging, and multi-omics datasets to improve disease prediction, biomarker discovery, and patient stratification. AI approaches, including random forest, support vector machine, convolutional neural networks, and XGBoost, together with microbiome analysis platforms such as QIIME 2 and multi-omics integration tools including mixOmics and MOFA+, are highlighted for their potential in precision neurology. Collectively, integrating GBA biology with AI-driven analytics provides promising opportunities for early diagnosis, personalised therapeutic strategies, and precision medicine to address the growing burden of neurodegenerative disorders.

## 1 Introduction

The rapidly increasing global health burden of neurodegenerative disorders, especially Alzheimer’s disease (AD) and Parkinson’s disease (PD), is a major concern because these are the most prevalent and debilitating conditions (Wang et al., 2024). Sharma et al. (2025) reported that characteristics such as progressive neuronal loss, accumulation of misfolded proteins, and irreversible decline in cognitive and motor functions are associated with AD and PD. AD is associated with memory impairment and cognitive dysfunction and is primarily characterised by increased extracellular amyloid-β (Aβ) plaques and intracellular Tau neurofibrillary tangles (Abdulkhaliq et al., 2026; Upadhyay et al., 2026). Similarly, PD is characterised by degeneration of dopaminergic neurons in the substantia nigra (SN) and α-synuclein (α-syn) aggregation, which ultimately leads to motor impairments such as tremors, rigidity, and bradykinesia (Pitton Rissardo et al., 2025). Loh et al. (2024) suggested that AD and PD are not solely brain-centric disorders because of alterations in peripheral systems, especially the gastrointestinal system, which plays a major role. The gut-brain interaction has been recognised as a critical interface linking the central nervous system with the immune system through neural, immune, endocrine, and metabolic pathways (Barbu et al., 2025). The microbial communities in the gut influence host metabolism, immune response, and neurochemical signalling, thereby contributing to brain health and disease (O’Riordan et al., 2025).

Reduced microbial diversity, altered taxonomic composition, depletion of beneficial short-chain fatty acids (SCFAs), which are produced by bacteria, and enrichment of pro-inflammatory microbial taxa are major characteristics of gut dysbiosis in AD and PD (Fusco et al., 2023; Wu et al., 2025). These alterations compromise the integrity of the intestinal barrier, leading to increased intestinal permeability, often referred to as “leaky gut” (Aleman et al., 2023). The microbial products, such as lipopolysaccharides (LPS), that translocate into the systemic circulation trigger immune activation and chronic inflammation, which in turn leads to disruption of the blood-brain barrier (BBB), activation of microglia, and promotion of neuroinflammatory cascades that contribute to neuronal damage and disease progression (Peng et al., 2021; Kalyan et al., 2022). Moreover, the microbial metabolites and amyloid-like proteins produced by the gut bacteria may influence protein misfolding and aggregation processes involving Aβ in AD and α-syn in PD (Jain, 2024; Park et al., 2025). Furthermore, the vagus nerve is a key neural conduit supporting gut-brain interaction, particularly in PD, where pathological propagation of α-syn from the enteric nervous system to the central nervous system has been proposed (Luo et al., 2025).

Current research using high-throughput sequencing technologies and multi-omics approaches has enabled deeper insights into the complex interactions between the gut microbiome and host system (Chetty and Blekhman, 2024). Consequently, artificial intelligence (AI) and machine learning (ML) have reshaped the analysis of these large and complex datasets. AI-driven models facilitate early diagnosis, disease prediction, and patient stratification by identifying subtle patterns across multimodal data, including neuroimaging, biofluid biomarkers, genetic profiles, and digital health metrics (Zhou et al., 2025; Trasca et al., 2025). In this context, precision medicine represents a transformative approach to the management of AD and PD, aiming to tailor therapeutic strategies based on individual variability through the integration of multi-omics data, clinical phenotypes, and microbiome profiles (Molla and Bitew, 2024; Paul et al., 2025). Moreover, lifestyle modification is increasingly recognised as an essential component of personalised treatment strategies (Ho et al., 2020). Collectively, the interplay between the gut microbiome, neuroinflammation, and neurodegeneration underscores the importance of adopting a holistic and integrative framework in understanding AD and PD (Palanivelu et al., 2025). The convergence of microbiome research, computational biology, and precision medicine holds significant promise for developing novel diagnostic and therapeutic strategies aimed at early intervention and disease modification (Hadrich, 2018).

## 2 Gut-brain axis

The microbial communities comprising trillions of microorganisms, including bacteria, viruses, fungi, and archaea, play a fundamental role in maintaining homeostasis, which influences brain function through metabolic signalling, immune modulation, neurotransmitter production, and maintenance of intestinal barrier integrity (Hou et al., 2022; Colella et al., 2023; O’Riordan et al., 2025; Upadhyay et al., 2025). Hence, interconnected neural, immune, endocrine, and metabolic signalling pathways and alterations in microbial composition are the primary means of gut-brain communication (Solanki et al., 2023; Zheng et al., 2023; Shen et al., 2025). Yu et al. (2020) and Faraji et al. (2025) suggested that sensory neurons in the gut detect microbial metabolites and inflammatory signals, transmitting information to the central nervous system. In addition, growing evidence suggests the pathological misfolding of α-syn in PD may originate in the enteric nervous system and propagate to the brain via vagal pathways (Kim et al., 2019; Xiang et al., 2024). Emerging evidence suggests that gut-brain interactions may contribute to AD pathogenesis and progression; however, current evidence does not support the generalization that AD pathology originates in the gastrointestinal tract. This distinction is important because prodromal gastrointestinal involvement is more firmly established in PD (**Figure 1**).

**Figure 1:**
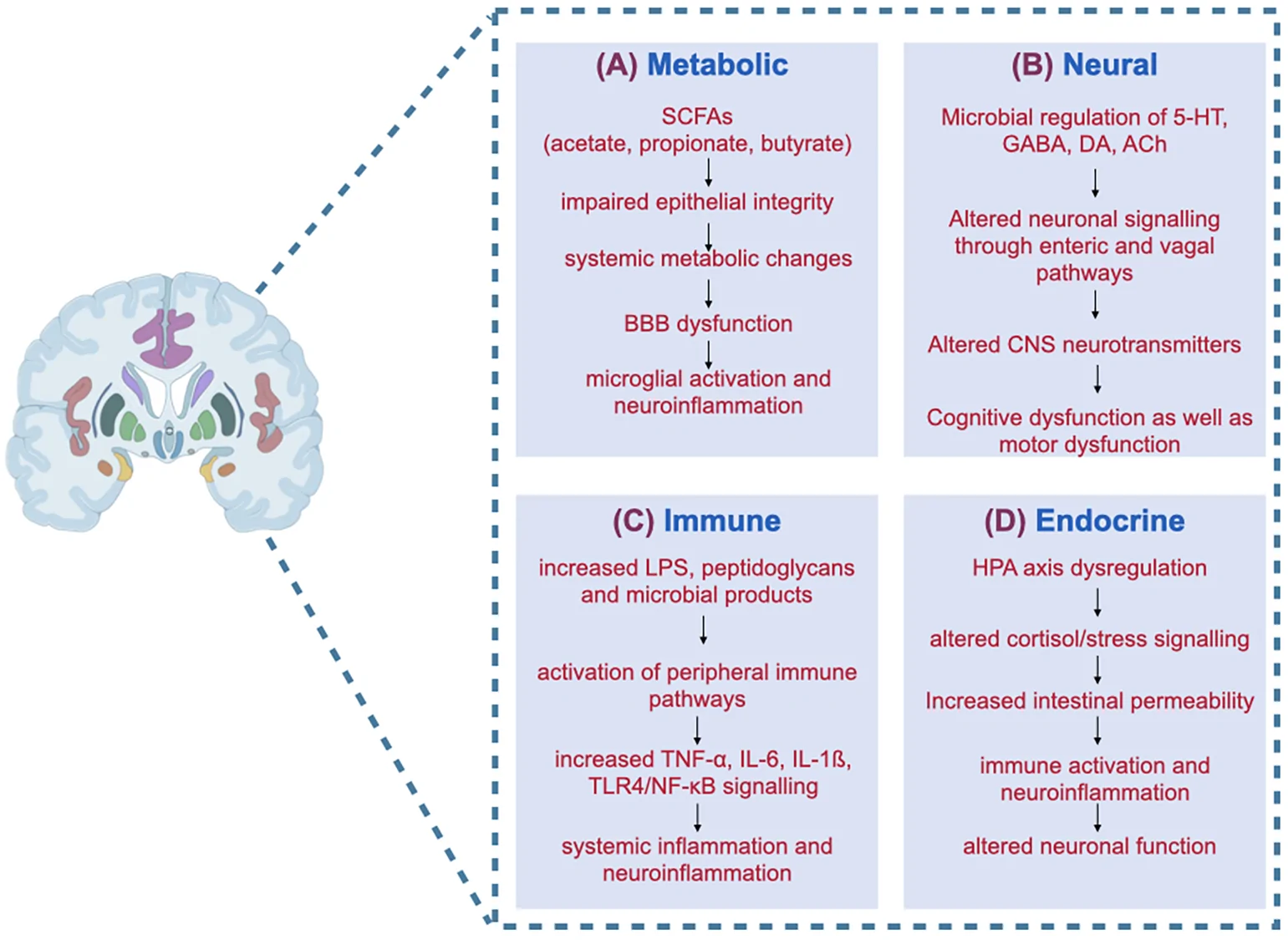
Major gut-brain axis signalling pathways linking gut microbial alterations with neurological dysfunction. **(A)** Metabolite signalling mediated primarily by microbial short-chain fatty acids (SCFAs), including acetate, propionate, and butyrate, and their effects on intestinal epithelial integrity, systemic metabolism, blood-brain barrier (BBB) function, and neuroinflammation. **(B)** Neurotransmitter-mediated signalling involving microbial regulation of serotonin (5-HT), γ-aminobutyric acid (GABA), dopamine (DA), and acetylcholine (ACh), with downstream effects on enteric, vagal, and central neuronal signalling. **(C)** Immune-mediated signalling involving microbe-associated molecules such as lipopolysaccharides (LPS) and peptidoglycans, activation of peripheral immune pathways, and increased pro-inflammatory signalling through TNF-α, IL-6, IL-1β, and TLR4/NF-κB. **(D)** Hypothalamic-pituitary-adrenal (HPA) axis signalling, whereby altered stress and cortisol responses may contribute to increased intestinal permeability, immune activation, neuroinflammation, and altered neuronal function. Collectively, these pathways illustrate the bidirectional communication between the gut microbiota and the central nervous system and their potential contribution to neurodegenerative disease.

The microbial products and immune signals are regulated by the intestinal epithelium, which functions as a barrier (Yoo et al., 2020; Socała et al., 2021). Hence, dysbiosis in the microbiome can disrupt this intestinal epithelium, which leads to increased intestinal permeability, often referred to as leaky gut, resulting in the entry of LPS and peptidoglycans into the systemic circulation (Di Vincenzo et al., 2024). The leaky gut caused by systemic inflammation can disrupt the BBB, resulting in microglial activation and neuroinflammation, both hallmark features of AD and PD (Bayraktaroglu et al., 2025). An important mechanism linking the gut microbiota to brain function is metabolite signalling, whereby microorganisms ferment dietary fibres and in turn produce short-chain fatty acids (SCFAs), primarily acetate, propionate, and butyrate (Facchin et al., 2024). Metabolites play a significant role in the maintenance of intestinal integrity, regulation of immune response, and modulation of neuronal activity. Hence, butyrate acts as a histone deacetylase inhibitor and influences gene expression associated with neuroprotective and anti-inflammatory pathways (Salvi and Cowles, 2021). In both AD and PD, a reduction in SCFA-producing bacteria has been reported, which leads to diminished production of these beneficial metabolites. In addition, a reduction in SCFA levels may contribute to increased intestinal permeability, systemic inflammation, and altered microglial activation within the brain (Qian et al., 2022; Duan et al., 2024). 

The gut microbiota is influenced by neurotransmitters and neuromodulators, which include serotonin, dopamine precursors, gamma-aminobutyric acid (GABA), and acetylcholine (Mhanna et al., 2024). These neurotransmitters influence mood, cognition, and motor function either directly through neural pathways or indirectly through immune and metabolic signalling (Akram et al., 2024). The gut-brain axis also influences the protein misfolding typically observed in neurodegenerative diseases, as the gut microbiota produces amyloid-like proteins that structurally resemble pathological proteins found in neurodegenerative diseases (Wei et al., 2022). Hence, these microbial amyloids can stimulate immune responses and potentially promote cross-seeding of host proteins and aggregation processes such as Aβ accumulation in AD or α-syn aggregation in PD (Sampson, 2025). Stress-related hormones, such as cortisol, can alter the gut microbial composition and intestinal barrier function, suggesting that the interaction between the gut microbiota and the hypothalamic-pituitary-adrenal (HPA) axis regulates the physiological stress response (Rusch et al., 2023; Pires et al., 2024). Furthermore, microbial metabolites can influence HPA axis activity, which in turn affects stress resilience and cognitive function; dysregulation of this system is linked with neuroinflammation and neurodegeneration (Ataei et al., 2026). Factors such as diet, lifestyle, and environment play a major role in the gut microbial ecosystem and consequently gut-brain interaction (Berding et al., 2021). The microbial populations produce neuroprotective metabolites from dietary patterns such as those rich in fibres, polyphenols, and fermented food (Rudrapal et al., 2026). Subsequently, both PD and AD tend to influence neuroinflammation, protein aggregation, metabolic signalling, and neurotransmitter balance (Jin et al., 2026; Chen et al., 2025). Therefore, the underlying mechanism which facilitates gut-brain interaction may lead to novel therapeutic strategies, including probiotics, prebiotics, dietary modification, and faecal microbiota transplantation (Ullah et al., 2023; Ugwu et al., 2025).

## 3 Gut microbiota dysbiosis in AD and PD

In neurological conditions such as AD and PD, dysbiosis commonly involves reduced microbial diversity, altered *Firmicutes/Bacteroidetes* ratios, and depletion of SCFA-producing bacteria such as *Faecalibacterium, Roseburia*, and *Eubacterium, thereby* enriching pro-inflammatory taxa and increasing gut permeability (Fusco et al., 2023; Zhao et al., 2023), as shown in **Table 1**. Reduced abundance of SCFA-producing bacteria, particularly butyrate-producing microorganisms, may decrease SCFA availability and impair intestinal tight-junction integrity, leading to increased intestinal permeability; conversely, disruption of the intestinal barrier may further contribute to microbial dysbiosis and altered metabolite production (Silva et al., 2020; Kalkan et al., 2025; Ravi et al., 2025). The translocation of bacterial endotoxins such as lipopolysaccharide (LPS) activates toll-like receptor 4 (TLR4), triggers nuclear factor kappa B (NF-κB)-mediated inflammatory cascades, drives microglial activation, and elevates cytokines [tumour necrosis factor alpha (TNF-α), interleukin 1 beta (IL-1β), and interleukin 6 (IL-6)]; the disruption of epithelial barriers exacerbates amyloidogenesis in AD and accelerates α-syn pathology in PD (Batista et al., 2019; Page et al., 2022). In addition, activation of the NLR family pyrin domain containing 3 (NLRP3) inflammasome provides an important downstream mechanism linking microbial and metabolic stress to neuroinflammation (Demirtaş et al., 2023). Gut barrier dysfunction and increased exposure to microbial products can provide inflammatory priming through TLR4/NF-κB signalling, followed by NLRP3 inflammasome activation and caspase-1-mediated maturation of IL-1β and interleukin 18 (IL-18) (Yang et al., 2023). In AD, NLRP3 activation has been associated with Aβ-induced microglial activation, impaired inflammatory regulation, and progression of neuroinflammation (Jung et al., 2022). Similarly, in PD, α-synuclein aggregates can activate NLRP3-dependent inflammatory responses in microglia, thereby contributing to neuronal injury and disease progression (Li et al., 2021). Thus, interactions amongst gut-derived microbial products, TLR4/NF-κB signalling, and NLRP3 inflammasome activation may represent a common inflammatory pathway linking intestinal dysfunction with central nervous system (CNS) inflammation in AD and PD (Zheng et al., 2020). In particular, the gut microbiota in PD contains certain bacteria, such as *Escherichia coli*, which also produce functional amyloids, and these can cross-react with host proteins, promoting Aβ aggregation and α-synuclein misfolding, reinforcing the gut-origin hypothesis (Ghosh and Sinha, 2025). Additionally, the gut microbes synthesise the key neuroactive molecules, including serotonin (5-HT), dopamine precursors, GABA, and acetylcholine, influencing central neurotransmission via vagal and immune pathways, and early vagus nerve involvement in PD supports a gut-to-brain transmission route (Chen et al., 2021; Qu et al., 2024). Decreased *Bifidobacterium* and *Firmicutes*, increased *Bacteroidetes* and pro-inflammatory *Proteobacteria*, enrichment of *Escherichia/Shigella, and* loss of butyrate producers have been reported in many clinical and preclinical studies and have been correlated with higher inflammatory markers and more severe cognitive decline (Hou et al., 2022; Liang et al., 2024; Dissanayaka et al., 2024). Furthermore, AD progression via Aβ aggregation happens due to microbial metabolites, which lead to mitochondrial dysfunction, oxidative stress, and a shift in tryptophan metabolism towards the kynurenine pathway, yielding neurotoxic metabolites such as quinolinic acid (Missiego-Beltrán and Beltrán-Velasco, 2024). In PD, gastrointestinal symptoms such as constipation and altered motility often precede motor signs by years, indicating early enteric nervous system involvement, and typical microbiota changes include reduced *Prevotella*, decreased SCFA producers, increased *Enterobacteriaceae*, and elevated *Akkermansia*; lower *Prevotella* is associated with reduced mucin and impaired barrier function, whilst higher *Enterobacteriaceae* correlates with greater postural instability and gait disturbance (Yang et al., 2019; Zhang et al., 2023a). Germ-free α-syn-overexpressing mice exhibit attenuated motor deficits that reappear after colonisation with PD patient microbiota, implicating a causal role for the microbiota, and SCFAs may have context-dependent effects, supporting homeostasis in health but contributing to inflammation in disease (Sampson et al., 2026; Kang et al., 2021; Morais et al., 2024). Vaziri et al. (2025) suggested that gut microbiota dysbiosis is a peripheral contributor to the pathogenesis of AD and PD, though with distinct disease-specific trajectories. Early gastrointestinal involvement in PD provides evidence of prion-like gut-to-brain propagation (Schmitt et al., 2023), whereas AD is closely linked to metabolic dysfunction, insulin resistance, and systemic inflammatory priming (de la Monte, 2017). The altered microbial composition leads to reduced SCFA production, increased intestinal permeability, systemic inflammation, and microbial amyloid cross-seeding, causing neuroinflammatory cascades and pathological protein aggregation (Czaj et al., 2026). Recognition of these mechanisms has prompted exploration of microbiome-targeted therapies, including probiotics and synbiotics (*Lactobacillus* and *Bifidobacterium*), which improve cognitive scores and reduce inflammatory markers in AD and alleviate constipation and potentially systemic inflammation in PD (Jafari et al., 2025; Páez-García et al., 2025); prebiotics and dietary fibre to promote SCFA production and restore epithelial barrier integrity (Choe, 2025); faecal microbiota transplantation, which shows preliminary symptomatic benefits in small PD cohorts but still requires controlled trials (De Sciscio et al., 2025); postbiotics and microbial metabolite modulation, such as direct butyrate administration or engineered bacteria for precision interventions (Thorakkattu et al., 2022); and precision microbiome medicine that integrates metagenomics, metabolomics, and host genetics to enable personalised modulation strategies (Yang et al., 2025). Future longitudinal and mechanistic studies combining multi-omics profiling, advanced neuroimaging, and detailed clinical phenotyping are essential to establish causality, identify robust microbial biomarkers, and fully realise microbiome-based therapeutics as adjunctive strategies for neuroprotection and disease modification in these devastating neurodegenerative disorders (Babu and Snyder, 2023).

**Table 1:** Comparative gut microbiota alterations in AD and PD.

Factors	Alzheimer’s disease	Parkinson’s disease
Microbial Diversity	Reduced	Reduced
SCFA-Producing Bacteria	Decreased (*Faecalibacterium, Roseburia*)	Decreased (*Prevotella*, *Blautia*)
Pro-inflammatory Taxa	Increased *Proteobacteria*	Increased *Enterobacteriaceae*
Intestinal Permeability	Increased	Increased
Microbial Amyloid Role	Promotes Aβ aggregation	Promotes α-synuclein aggregation
Primary CNS Outcome	Cognitive decline	Motor and non-motor symptoms
Gut Symptoms	Mild/secondary	Early constipation (prodromal)
Key Pathological Protein	Amyloid-β, Tau	α-Synuclein

Although numerous studies have reported associations between gut microbial alterations and neurodegenerative disease, the strength of evidence differs substantially according to study design (Liu et al., 2024). In addition, differences in age, sex, geographical location, dietary patterns, disease stage, medication exposure, sample collection, sequencing platforms, and bioinformatic pipelines can contribute to variation between cohorts (Kabir et al., 2025). Bostick et al. (2022) have demonstrated that manipulation of the gut microbiota can influence neuroinflammation, protein aggregation, and neurological phenotypes. Similarly, Morais et al. (2024) observed that germ-free α-synuclein-overexpressing mice show attenuated motor abnormalities that can be restored following colonisation with microbiota from individuals with PD, providing experimental support for a causal contribution of the microbiota. SCFAs, particularly butyrate, have been associated with maintenance of intestinal barrier integrity and immune homeostasis, but their effects may depend on concentration, microbial context, host metabolic state, and disease stage (Savitz, 2021; Zhang et al., 2023b). These findings suggest that gut-brain axis (GBA)-mediated mechanisms are likely to involve interactions amongst multiple microbial, metabolic, immune, and host factors rather than a single pathogenic microbial component (Gao et al., 2020). Therapeutic evidence is currently less mature than evidence for disease-associated microbial alterations such as probiotic, prebiotic, dietary, postbiotic, and faecal microbiota transplantation approaches (Ji et al., 2023). Preliminary faecal microbiota transplantation (FMT) studies in PD indicate feasibility and potential symptomatic benefit, but small cohorts and limited follow-up prevent conclusions regarding long-term disease modification. Thus, current evidence supports the gut-brain axis as a biologically relevant contributor to neurodegenerative disease whilst highlighting the need for longitudinal human studies, mechanistic validation, standardised microbiome methodologies, and adequately powered controlled clinical trials (Hamilton et al., 2024; Nabil et al., 2025).

## 4 AI and ML diagnostic approaches

In neurodegenerative disorders, particularly in AD and PD, AI and ML have emerged as transformative diagnostic tools (Zhang et al., 2025). These complex, progressive, and multifactorial disorders have often been diagnosed only after significant neuronal damage has occurred because traditional diagnostic approaches depend on clinical symptoms, neuropsychological testing, and imaging findings that typically reflect late-stage pathology (Elahi and Miller, 2017; Wahul et al., 2025). Parvin et al. (2025) suggested that AI and ML approaches enable early detection by identifying subtle and high-dimensional patterns across multimodal datasets, including neuroimaging, biofluid biomarkers, genomics, metabolomics, and digital health metrics. Johnson et al. (2021) and Martel et al. (2026) suggested that by leveraging large-scale computational modelling, AI might enhance diagnostic precision, risk stratification, and disease monitoring, which offers potential pathways towards preclinical identification and personalised intervention. Although many current AI applications in AD and PD focus on neuroimaging and clinical biomarkers, these modalities can be combined with GBA-derived features to provide a more comprehensive representation of disease heterogeneity (Liu et al., 2025). Microbiome composition and microbial metabolites could be integrated with MRI or PET measures to investigate whether specific peripheral biological signatures are associated with patterns of brain atrophy, neuroinflammation, amyloid deposition, or dopaminergic dysfunction (Jia et al., 2026). Such multimodal integration may allow AI models to identify relationships between peripheral GBA alterations and central neurological phenotypes that would remain undetected when individual data types are analysed separately (Huang and Shu, 2025). Imaging modalities, including structural magnetic resonance imaging (MRI), functional MRI (fMRI), positron emission tomography (PET), and dopamine transporter single-photon emission computed tomography (DAT-SPECT), provide rich datasets suitable for deep learning analysis (Palermo et al., 2021; Jimenez-Mesa et al., 2023). Raza et al. (2025) also suggested that diagnostic methods such as convolutional neural networks (CNNs) can detect subtle hippocampal atrophy, cortical degradation, and alterations in network connectivity in early AD. With the recent advances, the PET-based ML classifiers differentiate amyloid-positive from amyloid-negative individuals with high sensitivity and specificity. Hence, the ML algorithms are applied to DAT-SPECT for PD and with the help of diffusion tensor imaging, identification of nigrostriatal degeneration and white matter microstructural change can be carried out. These particularly help in differentiating between idiopathic PD and atypical Parkinsonian syndromes, improving early diagnostic accuracy where clinical overlap is common (Kim et al., 2021; Gujral, 2024).

The increasing use of high-throughput microbiome sequencing and multi-omics technologies has created new opportunities to investigate the gut-brain axis at multiple biological levels (Zhong et al., 2026). Microbiome analysis platforms such as QIIME 2 provide reproducible workflows for processing sequencing data, including quality assessment, feature generation, taxonomic classification, and microbial diversity analysis (Estaki et al., 2020). In the context of AD and PD, such approaches can facilitate the identification of disease-associated alterations in microbial community structure and composition and their relationships with clinical phenotypes (Baldini et al., 2020). However, microbiome-derived signatures can be influenced by dietary habits, medication exposure, age, geographical variation, sample-processing procedures, sequencing platforms, and bioinformatic pipelines (Wang et al., 2019; Huang et al., 2026). Morabito et al. (2025) reported that tools such as mixOmics support multivariate integration and feature selection across heterogeneous omics datasets, whereas MOFA+ uses latent-factor modelling to identify coordinated patterns of variation across multiple molecular layers. Within GBA research, these approaches may facilitate the integration of microbiome profiles with metabolomics, transcriptomics, proteomics, inflammatory biomarkers, and clinical phenotypes to identify coordinated molecular signatures associated with AD or PD (Ni et al., 2026). Therefore, QIIME 2, mixOmics, and MOFA+ should be considered enabling analytical frameworks for GBA research rather than established diagnostic tools, and their clinical relevance requires independent validation and prospective evaluation (Licata et al., 2025).

Moreover, random forests, support vector machines, and gradient boosting are ML algorithms that can be integrated with cerebrospinal fluid (CSF) markers, including amyloid-β42, total Tau, and phosphorylated Tau in AD, and α-synuclein species in PD (Winchester et al., 2023; Gaeta et al., 2024). Amongst gradient-boosting methods, XGBoost (Extreme Gradient Boosting) is particularly applicable to structured biomedical datasets because it can model non-linear relationships and interactions amongst multiple predictors whilst incorporating regularisation to reduce overfitting (Pellegrino et al., 2023). In AD and PD, XGBoost can potentially integrate clinical characteristics, genetic variants, inflammatory biomarkers, microbiome-derived features, and metabolomic profiles for disease classification, risk prediction, and patient stratification (Wang et al., 2026). Within GBA-focused research, XGBoost may be particularly useful for identifying complex associations between microbial taxa, microbial metabolites, inflammatory mediators, and neurological phenotypes that may not be captured by conventional statistical approaches (Chen et al., 2025). Feature-importance and model-explainability approaches can further assist in identifying variables that contribute to model predictions (Veledar et al., 2025). Therefore, high predictive performance within a development cohort should not be interpreted as evidence of clinical validity without robust external and prospective validation. Multivariate ML models combine demographic variables with biomarker panels and usually improve early risk classification compared with single-marker approaches (Jing et al., 2024). Microbiome-based machine-learning models can subsequently use microbial diversity indices, taxonomic abundance, and metabolite profiles as predictive features for cognitive decline in AD or prodromal motor manifestations in PD (Romano et al., 2025). Integration of these microbiome-derived variables with host molecular and clinical data may provide a more comprehensive representation of GBA-associated disease heterogeneity (Taj et al., 2025). However, the predictive value of such models depends on cohort size, feature reproducibility, appropriate control of confounding variables, and validation in independent populations (Zacharias et al., 2022; Hamilton et al., 2024). Yetgin (2025) suggested that a deep neural network can analyse high-dimensional datasets derived from genome-wide transcriptomics, epigenomics, and metabolomics. In addition, integrating genetic risk factors such as *APOE* with ML models in AD might improve predictive accuracy for disease progression (Al-Hammadi et al., 2025). Consequently, in PD, the ML algorithms analyse genetic variants alongside transcriptomic and inflammatory gene expression to identify high-risk individuals (Su et al., 2020).

Currently, the use of AI-based diagnostic wearable sensors and smartphone-based motion tracking systems helps to generate continuous data on tremor amplitude, bradykinesia, gait instability, and motor fluctuation (Sun et al., 2024). Hence, analysis using recurrent neural networks and long short-term memory models can detect longitudinal motor patterns, identify subtle abnormalities early, and enable remote monitoring (Yoon et al., 2023). Alongside, Zozuk et al. (2025) suggested that in AD, the assessment of speech patterns, semantic errors, pause frequency, and linguistic diversity to detect early cognitive decline can be analysed via natural language processing techniques. In addition, AI-based voice analysis provides a non-invasive, scalable screening tool with potential for community-level implementation (Krautz et al., 2025; Twala, 2025). Hence, the overall AI and ML diagnostic approaches (**Table 2**) are redefining early detection paradigms in AD and PD by integrating multimodal biological and digital data into predictive frameworks (Kumar et al., 2024; Mohammed and Malhotra, 2025). Hence, with validation, standardised datasets, and ethical implementation strategies, AI-driven diagnostics might become significant for precision neurology in the coming years (Baklola et al., 2025; Fahim et al., 2025). Integrating microbiome-derived features with host multi-omics, neuroimaging, and longitudinal clinical data may therefore provide a GBA-centred framework for AI-assisted biomarker discovery and patient stratification (Kedari et al., 2026). However, prospective validation is required before such models can be translated into routine clinical practice (Knapen et al., 2026). Microbiome-based models present additional challenges because microbial abundance data are compositional and are strongly influenced by diet, medication, geography, and sample-processing procedures (Ece et al., 2026). Similarly, multimodal models integrating imaging, omics, and clinical data may achieve high predictive performance whilst remaining difficult to interpret biologically. Therefore, model accuracy within a development cohort should not be equated with clinical validity (Marouf et al., 2025).

**Table 2:** Overview of AI and ML diagnostic approaches in AD and PD.

Data modality	Algorithms commonly used	Application in AD	Application in PD	Key advantages	Major limitations
Neuroimaging (MRI, PET, DAT-SPECT)	CNN, SVM, random forest	Detect hippocampal atrophy; classify amyloid positivity	Identify dopaminergic loss; differentiate parkinsonism	High diagnostic accuracy	Expensive; requires large datasets
Biofluid Biomarkers (CSF, Plasma)	random forest, gradient boosting	Predict Aβ/tau pathology	Assess α-synuclein burden	Minimally invasive	Inter-laboratory variability
Multi-Omics Integration	deep neural networks, autoencoders	Genetic risk and progression modelling	Molecular risk stratification	Comprehensive profiling	Computational complexity
Microbiome-Based Models	SVM, random forest	Dysbiosis-based cognitive prediction	Early PD classification	Non-invasive; emerging	Standardisation challenges
Digital Biomarkers	RNN, recurrent neural network; LSTM, long short-term memory; NLP, natural language processing	Speech-based cognitive assessment	Gait and tremor monitoring	Remote and continuous tracking	Device variability; validation required

## 5 Precision medicine

Precision medicine provides a framework for moving from population-level descriptions of neurodegenerative disease towards biologically informed patient stratification (Rossi et al., 2023). Within the gut-brain axis, this approach requires consideration of interactions amongst host genetics, gut microbial composition, microbial metabolites, immune responses, gastrointestinal function, lifestyle, and central nervous system phenotypes (Chakrabarti et al., 2022). Rather than assuming that a single microbial alteration or therapeutic intervention will produce similar effects across all patients, GBA-informed precision medicine seeks to identify patient subgroups with distinct biological and clinical profiles and to determine whether these differences can guide prevention, diagnosis, or treatment (Strafella et al., 2018; Velikic et al., 2025). Substantial heterogeneity in the clinical presentation, rate of progression, molecular pathology, and treatment response in AD and PD often necessitates traditional therapeutic regimens which primarily provide symptomatic relief, often without addressing patient-specific disease drivers (Duara and Barker, 2022). Hence, Wang and Wang (2023) and Khare et al. (2026) reported that precision medicine aims to stratify patients into biologically meaningful subgroups and tailor interventions according to individual risk profiles and mechanistic pathways (as shown in **Table 3**). In addition, in AD, genetic profiling plays a central role in precision approaches because of variants such as *APOE* ϵ4, which significantly influence disease risk, age of onset, and amyloid burden (Raulin et al., 2022). Integrating genomic data with plasma biomarkers (Aβ42/40 ratio, phosphorylated Tau), neuroimaging findings, and cognitive phenotyping enables more accurate disease staging and risk prediction (Um et al., 2025). Molecular subtyping based on inflammatory signatures, metabolic dysfunction, or Tau-dominant pathology allows targeted interventions, including anti-amyloid therapies, anti-inflammatory strategies, or metabolic modulation (Hein et al., 2026). Precision frameworks also incorporate vascular risk factors, insulin resistance, and lipid metabolism profiles, acknowledging the multifactorial nature of AD pathogenesis (Berkowitz et al., 2018). From a GBA perspective, these established AD risk and biomarker profiles could be complemented by microbiome and metabolomic information (Vaziri et al., 2025). Integration of gut microbial composition with inflammatory markers, microbial metabolites, *APOE* genotype, plasma Aβ and phosphorylated Tau, metabolic phenotypes, and neuroimaging may help determine whether distinct GBA-associated biological subtypes exist within the broader AD population (Hammond et al., 2023). However, microbiome composition is strongly influenced by diet, medication, age, geography, and disease stage; therefore, such signatures require longitudinal validation before they can be used to guide individual-level risk prediction or treatment selection (Tegegne and Savidge, 2025).

**Table 3:** Precision medicine strategies in AD and PD.

Component	Application in Alzheimer’s disease	Application in Parkinson’s disease	Clinical benefit	Challenges
Genetic Profiling	*APOE*-based risk stratification	*LRRK2*, *SNCA*, and *GBA* mutation-based subtyping	Early risk prediction	Genetic counselling needs
Biomarker-Guided Therapy	Aβ and tau-targeted interventions	α-synuclein and inflammatory profiling	Mechanism-based treatment	Biomarker variability
Multi-Omics Integration	Molecular endotyping	Pathway-specific patient clustering	Personalised drug selection	High computational demand
Microbiome Stratification	Dysbiosis-linked cognitive decline	Gut-driven PD phenotypes	Non-invasive personalisation	Standardisation gaps
Lifestyle Personalisation	Diet, vascular risk control	Exercise and gut-focused interventions	Holistic disease modulation	Patient adherence

Mutations in *LRRK2, SNCA*, and *GBA* define distinct molecular subtypes with different progression trajectories and therapeutic targets; hence, inter-individual variability in motor symptoms, cognitive decline, autonomic dysfunction, and treatment response is characteristic of PD (Guo et al., 2025). Tanaka (2025) suggested that personalising dopaminergic therapy based on pharmacogenomic insights might minimise motor fluctuations and dyskinesias and that molecular profiling of α-synuclein species, neuroinflammatory markers, and mitochondrial dysfunction guides experimental disease-modifying strategies. Additionally, a patient stratification model further refines the intervention by incorporating the gut microbiome, especially in patients with prominent gastrointestinal dysfunction (Song and Shin, 2022). Combining genomics, transcriptomics, proteomics, metabolomics, epigenomics, and microbiome data through advanced computational platforms enables the identification of patient-specific molecular networks (Vitorino, 2024). Artificial intelligence-driven clustering algorithms can classify patients into endotypes characterised by shared biological mechanisms rather than solely clinical symptoms (Birkenbihl et al., 2023). PD may provide a particularly informative setting for GBA-informed precision medicine because gastrointestinal dysfunction, including constipation and altered gut motility, may occur before classical motor manifestations (Chiang and Lin, 2025). Integrating gastrointestinal phenotypes with microbiome composition, microbial metabolites, inflammatory biomarkers, genetic variants in *LRRK2, SNCA*, and *GBA*, α-synuclein-related biomarkers, and longitudinal motor and non-motor phenotypes could therefore help identify biologically distinct patient subgroups (Menozzi et al., 2026). Nevertheless, early gastrointestinal abnormalities do not by themselves establish a gut-origin mechanism, and the extent to which microbiome modification can alter long-term PD progression remains uncertain (Peters et al., 2026).

Lifestyle and environmental factors such as diet, physical activity, toxin exposure, sleep quality, and psychosocial stress influence neurodegenerative risk and progression (Madore et al., 2020). Hence, structured exercise regimens and microbiome-targeted nutritional interventions complement pharmacological strategies as personal lifestyle modifications (Prieto-González et al., 2025). Despite these advances, GBA-informed precision medicine remains largely investigational (Denaro et al., 2024). Major challenges include limited longitudinal cohorts, variability in microbiome composition between populations, difficulties in standardising microbiome and multi-omics measurements, high implementation costs, data-integration challenges, and ethical concerns surrounding genetic and health data (Mohr et al., 2024). Importantly, identifying a patient subgroup with a characteristic microbial or molecular profile does not necessarily demonstrate that targeted modification of that profile will improve clinical outcomes (Atreya and Neurath, 2024). Therefore, biomarker-based stratification must be accompanied by prospective studies demonstrating that patient selection based on GBA-related characteristics results in improved therapeutic efficacy or safety (Zhang et al., 2024a; Zhang et al., 2024b). Such evidence will be essential for transitioning GBA-informed precision medicine from a promising research framework to clinically actionable personalised care (Arafah et al., 2023).

## 6 Conclusion

The growing body of evidence supports an important role for the GBA and gut microbiota dysbiosis in the pathophysiology of AD and PD. However, the strength of evidence and degree of causality vary across mechanisms and study designs (**Figure 2**). In addition, advances in technologies such as AI and ML have further enhanced the ability to detect early disease signatures, as these approaches, combined with multi-omics profiling, provide a robust framework for understanding disease heterogeneity and identifying novel biomarkers. Interestingly, the emergence of precision medicine offers a promising avenue for tailoring therapeutic interventions based on individual patient profiles, incorporating genetic, molecular, and microbiome-related factors. Future research should focus on longitudinal and mechanistic studies to establish causal relationships and validate microbiome-based diagnostics and therapeutics. By bridging microbiology, neuroscience, and computational science, a more comprehensive and personalised approach to rapidly progressing AD and PD can be achieved, ultimately improving patient outcomes and quality of life.

**Figure 2:**
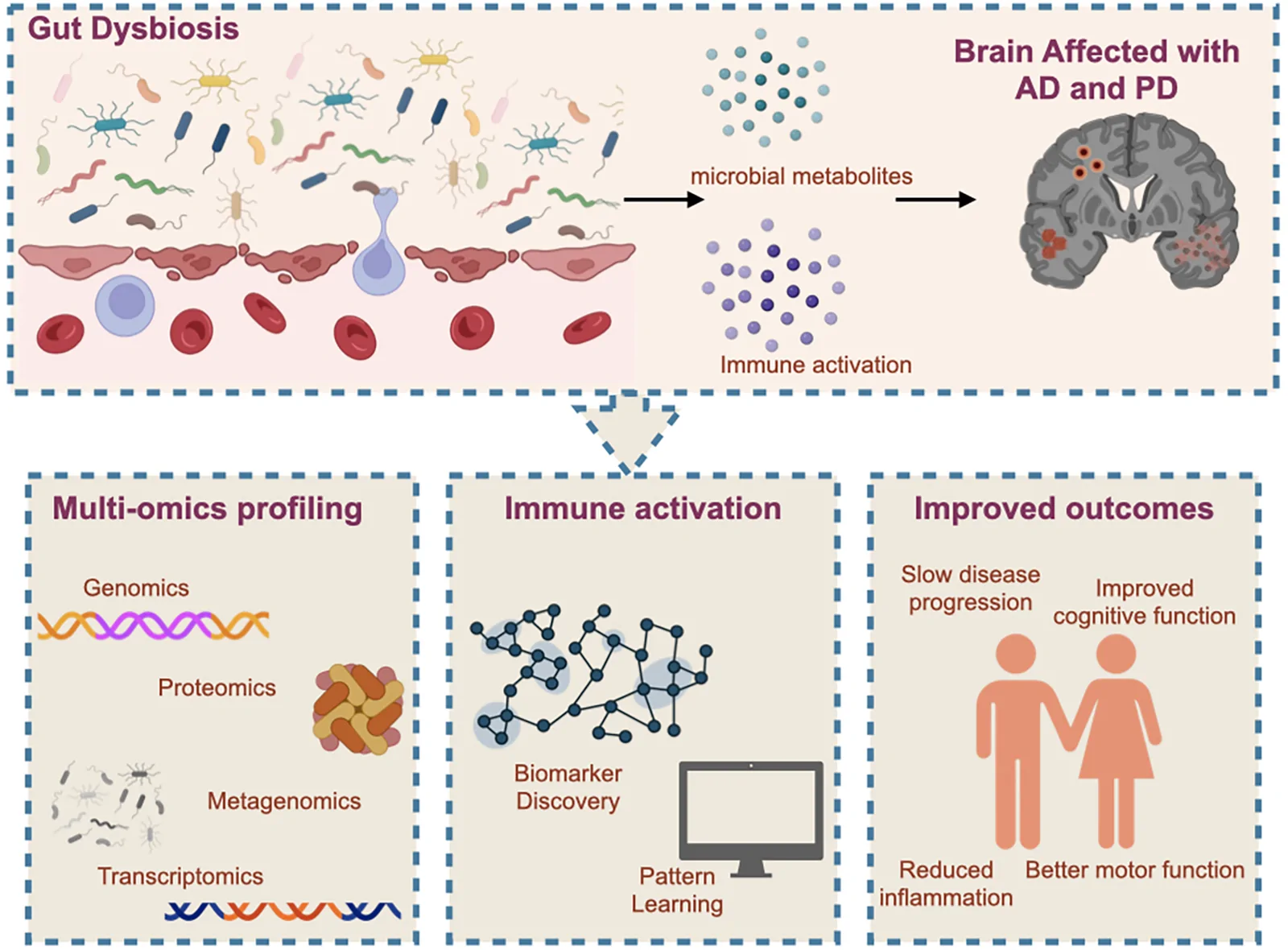
Integrative gut-brain axis framework linking gut dysbiosis, multi-omics profiling, computational analysis, and potential clinical outcomes in neurodegenerative diseases. Gut dysbiosis may alter microbial metabolites and stimulate immune activation, generating peripheral signals that can influence brain function and contribute to neurodegenerative pathology. Multi-omics profiling, including genomics, proteomics, metagenomics, and transcriptomics, can be used to characterise host-microbiome interactions and identify molecular signatures associated with disease. Computational approaches and pattern-learning methods may facilitate biomarker discovery and patient stratification by integrating heterogeneous biological and clinical datasets.
